# Development and validation of a prognostic model based on clinical laboratory biomarkers to predict admission to ICU in Omicron variant-infected hospitalized patients complicated with myocardial injury

**DOI:** 10.3389/fimmu.2024.1268213

**Published:** 2024-02-01

**Authors:** Xueying Yu, Xiaoguang Li, Shuai Xia, Tianyu Lu, Ming Zong, Chen Suo, Qiuhong Man, Lize Xiong

**Affiliations:** ^1^ Department of Clinical Laboratory, Shanghai Fourth People’s Hospital, School of Medicine, Tongji University, Shanghai, China; ^2^ Department of Thyroid, Breast and Vascular Surgery, Shanghai Fourth People’s Hospital, School of Medicine, Tongji University, Shanghai, China; ^3^ Key Laboratory of Medical Molecular Virology (Ministry of Education/National Health Commission/Chinese Academy of Medical Sciences, MOE/NHC/CAMS), Shanghai Institute of Infectious Disease and Biosecurity, School of Basic Medical Sciences, Shanghai Frontiers Science Center of Pathogenic Microbes and Infection, Fudan University, Shanghai, China; ^4^ Department of Epidemiology and Ministry of Education Key Laboratory of Public Health Safety, School of Public Health, Fudan University, Shanghai, China; ^5^ Shanghai Key Laboratory of Anesthesiology and Brain Functional Modulation, Clinical Research Center for Anesthesiology and Perioperative Medicine, Translational Research Institute of Brain and Brain-Like Intelligence, Shanghai Fourth People’s Hospital, School of Medicine, Tongji University, Shanghai, China

**Keywords:** omicron, myocardial injury, laboratory biomarkers, intensive care, prognostic model

## Abstract

**Aims:**

The aim of this study was to develop and validate a prognostic model based on clinical laboratory biomarkers for the early identification of high-risk patients who require intensive care unit (ICU) admission among those hospitalized with the Omicron variant of severe acute respiratory syndrome coronavirus 2 (SARS-CoV-2) and complicated with myocardial injury (MI).

**Methods:**

This single-center study enrolled 263 hospitalized patients with confirmed Omicron variant infection and concurrent MI. The patients were randomly divided into training and validation cohorts. Relevant variables were collected upon admission, and the least absolute shrinkage and selection operator (LASSO) was used to select candidate variables for constructing a Cox regression prognostic model. The model’s performance was evaluated in both training and validating cohorts based on discrimination, calibration, and net benefit.

**Results:**

Of the 263 eligible patients, 210 were non-ICU patients and 53 were ICU patients. The prognostic model was built using four selected predictors: white blood cell (WBC) count, procalcitonin (PCT) level, C-reactive protein (CRP) level, and blood urea nitrogen (BUN) level. The model showed good discriminative ability in both the training cohort (concordance index: 0.802, 95% CI: 0.716–0.888) and the validation cohort (concordance index: 0.799, 95% CI: 0.681–0.917). For calibration, the predicted probabilities and observed proportions were highly consistent, indicating the model’s reliability in predicting outcomes. In the 21-day decision curve analysis, the model had a positive net benefit for threshold probability ranges of 0.2 to 0.8 in the training cohort and nearly 0.2 to 1 in the validation cohort.

**Conclusion:**

In this study, we developed a clinically practical model with high discrimination, calibration, and net benefit. It may help to early identify severe and critical cases among Omicron variant-infected hospitalized patients with MI.

## Introduction

Since the emergence of the coronavirus disease 2019 (COVID-19) pandemic, various variants of the severe acute respiratory syndrome coronavirus 2 (SARS-CoV-2) have emerged, including the Omicron variant (1, 2). Although the Omicron variant may have lower virulence and pathogenicity than the previous Alpha and Delta variants (3–5), its remarkably high transmissibility and mild symptoms warrant attention (6–8). Therefore, the severity and mortality associated with the Omicron variant, especially among the elderly population, should not be overlooked (9, 10). Since early 2022, the rapid spread of the SARS-CoV-2 Omicron variant has triggered a surge in new cases across China, with the majority occurring in Shanghai (11, 12). Since March 2022, Shanghai has been facing the Omicron wave, witnessing a significant increase in severity and mortality rates, particularly among the elderly population, especially those with comorbidities (13, 14).

According to clinical case series, the incidence of myocardial injury (MI) during the SARS-CoV-2 epidemic varies from 7.2% to 36% (15–19), indicating its high prevalence in patients with COVID-19. Moreover, MI has been found to be significantly associated with the severity and mortality of COVID-19 (20). Several lines of evidence have demonstrated that MI is an independent risk factor for adverse outcomes. For example, a clinical study involving 41 COVID‐19 patients reported that 5 (12%) of them suffered from myocardial damage due to SARS-CoV-2 infection. Among these patients, four were treated in the intensive care unit (ICU), accounting for 31% of the total ICU admissions (19). Another clinical study involving 138 COVID‐19 patients also revealed that patients with MI during infection had a higher risk of deterioration, resulting in ICU admission (16). MI is a common complication observed in individuals infected with SARS-CoV-2, especially in elderly patients with multiple comorbid chronic diseases (17, 21–23). This association is important as it contributes to severe clinical manifestations and poor outcomes in COVID-19 patients.

Geriatric patients infected with the Omicron variant are a focus of clinical care as they have a higher risk of severity and mortality. Therefore, there is an urgent need in clinical care to stratify COVID-19 patients according to the presence of MI and to implement more aggressive treatment strategies. The aim of this study was to develop and validate a prognostic model to identify Omicron variant-infected hospitalized patients with MI who are at a higher risk of ICU admission, based on their age, gender, and clinical laboratory biomarkers.

## Methods

### Participants

**Figure 1:**
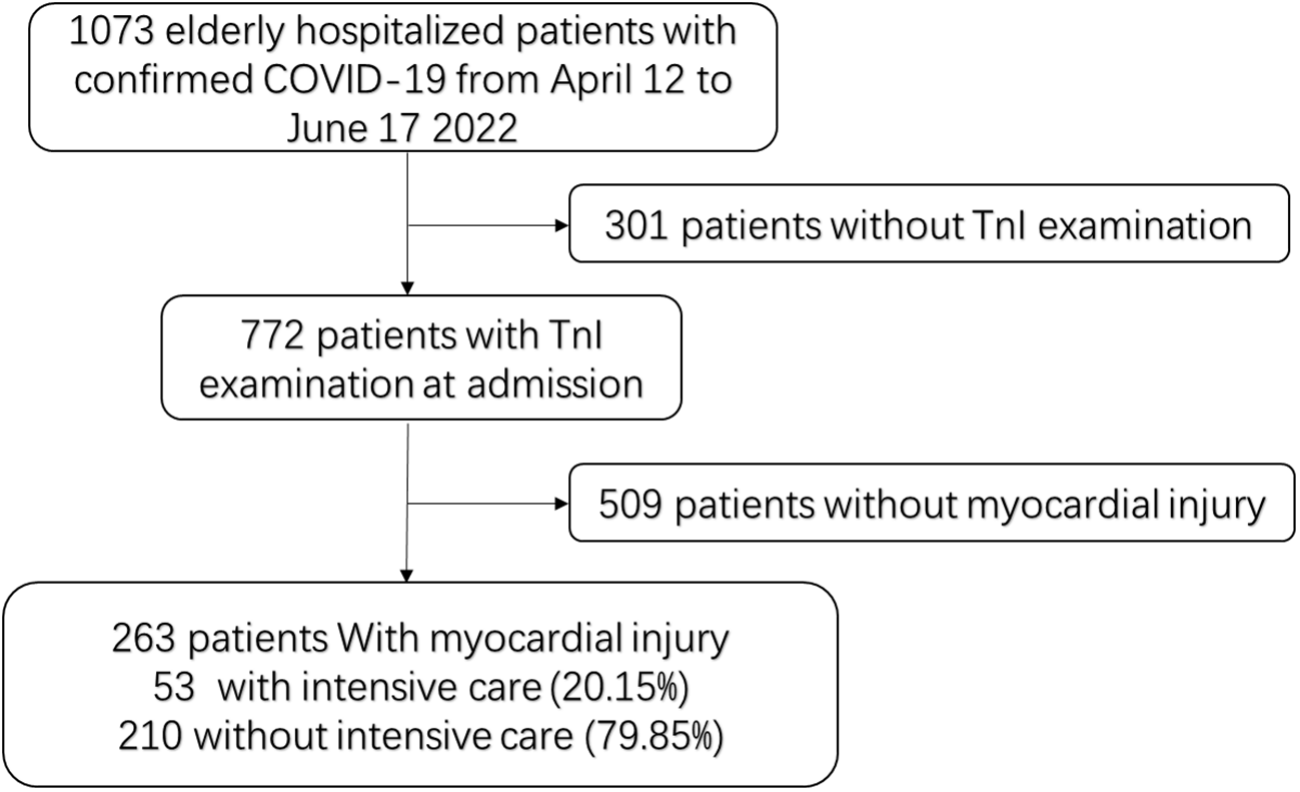
Flowchart of patient selection. TnI: Cardiac troponin I.

A single retrospective cohort study was carried out at Shanghai Fourth People’s Hospital from 12 April to 17 June 2022. We enrolled 263 Omicron variant-infected hospitalized patients aged >60 years with MI in our study. Of these, 53 patients with Omicron infection and MI were admitted to the ICU and categorized as the ICU group. The remaining 210 hospitalized patients with Omicron infection did not require ICU admission and formed the non-ICU group. Omicron variant infection was diagnosed and confirmed by positive real-time polymerase chain reaction results. Serum troponin I (TnI) was measured in patients who were admitted to the hospital within the first 24 h. MI was defined as a serum level of the cardiac biomarker TnI above 99% of the upper reference limit (15). The detailed inclusion process is shown in **Figure 1**.

The study was conducted following the 1975 Declaration of Helsinki (24), was approved by the Ethics Committee of the Fourth People’s Hospital of Tongji University (No. 2022097-001), and was published in the Chinese Clinical Trials Registry (CHiCTR2200065440). Written informed consent was waived by the ethics commission of the designated hospital for patients during the pandemic of the SARS-CoV-2 Omicron variant.

### Predictors and outcomes

Two researchers extracted demographic characteristics (age and gender) and clinical data (laboratory findings, treatments, and outcomes) from the hospital’s electronic medical records database for each patient admitted to the hospital.

Laboratory indicators, including data on (1) immune cells: white blood cell count (WBC, 10^9^/L), neutrophil count (NEUT, 10^9^/L), neutrophil percentage (NEUT%), lymphocyte count (LYM, 10^9^/L), lymphocyte percentage (LYM%), and hemoglobin (g/L) (2); inflammatory biomarkers: C-reactive protein (CRP, mg/L), procalcitonin (PCT, ng/mL), serum amyloid A (SAA, mg/L), and interleukin-6 (IL-6, pg/mL); and (3) liver function and myocardial enzyme spectrum: myoglobin (MYO, ng/L), creatine kinase-myocardial band (CK-MB, ng/L), N-terminal pro-B-type natriuretic peptide (NT-proBNP, pg/mL), total protein (TP, g/L), lactate dehydrogenase (LDH, U/L), aspartate aminotransferase (AST, U/L), alanine aminotransferase (ALT, U/L), estimated glomerular filtration rate (eGFR), blood urea nitrogen (BUN), and serum creatinine (sCr), were measured within the first 24 h of hospital admission, before transferring the patients to the ICU.

Patients received standard treatment according to the Diagnosis and Treatment Scheme of Pneumonia Caused by Novel Coronavirus of China (ninth version). Severe cases were defined as patients who had at least one of the following conditions: respiratory distress, low oxygen saturation, low PaO_2_/FiO_2_ ratio (PaO_2_ denotes partial pressure of oxygen in arterial blood and FiO_2_ denotes fraction of inspired oxygen), or progressive worsening symptoms with pulmonary imaging showing significant progression of lesions (>50%) within 24–48 h. Critical cases were defined as patients who met any of the following criteria: respiratory failure, shock, or organ failure requiring ICU admission (25). The outcome we focused on was ICU admission for the identification of severe or critical cases.

### Statistical analysis

We applied a 20% threshold for missing data, and variables with more than 20% missing values were excluded. For variables with less than 20% missing values, we used the R package “mice” to perform multiple imputation and generate reliable imputed values. Subsequently, one imputation result was selected for analysis. The eligible patients were divided into two cohorts: the training cohort, which included 70% of the patients, and the validation cohort, which included the remaining 30%. The least absolute shrinkage and selection operator (LASSO) was used for variable selection in the training cohort. To avoid overfitting and simplifying the model, we used LASSO regression to automatically screen features and shrink the coefficient estimates to zero. We also tuned the parameter selection in the LASSO model using minimum criteria. Based on the screening results of the LASSO methods, final predictors were used to establish Cox regression as the eventual prediction model.

The discrimination of the model was evaluated by the receiver operating characteristic (ROC) curve, the area under the receiver operating characteristic curve (AUROC), and the concordance index (C-index) with 95% confidence interval (95% CI). Calibration was assessed using a calibration curve that compared predicted probabilities with actual probabilities. The consistency between the predicted probabilities of the model and the actual proportions was evaluated by plotting calibration curves. Decision curve analysis (DCA) was used to demonstrate clinical net benefits under different threshold probabilities. The nomogram integrated multiple prediction indicators and presents the results graphically.

## Results

### Demographics and clinical characteristics

We included a total of 263 eligible hospitalized patients in our study (**Figure 1**). The median age of the eligible hospitalized patients was 87 (61–104) years, and 155 patients (58.94%) were women. Of these, 39 patients (73.58%) had severe disease courses. The patients had a high prevalence of comorbidities, with hypertension being the most common, followed by coronary artery disease and cerebrovascular disease. Among the patients, 53 (20.15%) had disease progression and required ICU admission (the ICU group), while the other 210 (79.85%) did not need ICU admission during their hospital stay (the non-ICU group). We systematically documented and analyzed the baseline characteristics of the patient cohort, including demographic and clinical laboratory biomarkers (**Table 1**). We found several variables that differed between the ICU and non-ICU groups: gender, leukocyte count, neutrophil count, lymphocyte count, D-dimer level, PCT level, CRP level, SAA level, MYO level, CK-MB level, NT-proBNP level, ALT level, eGFR, BUN level, and sCr level. Most of the patients were incompletely vaccinated (0 or 1 dose). There were no significant differences in the rates of full vaccination (2 or more doses) and incomplete vaccination (0 or 1 dose) between the ICU and non-ICU groups. Regarding the in-hospital treatment, the patients in the ICU group had a higher use of antiviral (plaxlovid) therapy, glucocorticoid therapy, transnasal high flow oxygen therapy, non-invasive ventilation, and invasive mechanical ventilation than the patients in the non-ICU group (**Table 1**).

**Table 1:** Demographics and clinical characteristics of 263 Omicron variant-infected hospitalized patients complicated with myocardial injury in the ICU group and non-ICU group.

	Patients, *n* (%)			
Characteristic	Total (*n* = 263)	ICU group (*n* = 53)	Non-ICU group (*n* = 210)	*p*-value
Demographics				
Age, median (range), years	87 (61–104)	88 (66–98)	87 (61–104)	0.42
Female	155 (58.94%)	39 (73.58%)	116 (55.24%)	0.015
Previous madical history				
Hypertension	184 (69.96%)	38 (71.70%)	146 (69.52%)	0.758
Coronary artery disease	117 (44.49%)	22 (41.51%)	95 (45.24%)	0.625
Cerebrovascular disease	92 (34.98%)	26 (49.06%)	66 (31.43%)	0.016
Diabetes	57 (21.67%)	12 (22.64%)	45 (21.43%)	0.848
Arrhythmia	47 (17.87%)	9 (16.98%)	38 (18.10%)	0.850
Neoplasm	21 (7.98%)	4 (7.55%)	17 (8.10%)	1.000
Chronic kidney disease	34 (12.93%)	11 (20.75%)	23 (10.95%)	0.057
Chronic pulmonary disease	6 (2.28%)	1 (1.89%)	5 (2.38%)	1.000
Heart failure	12 (4.56%)	2 (3.77%)	10 (4.76%)	1.000
Vaccination				
Unvaccinated/Partially (0/1 dose)	206 (78.33%)	41 (77.36%)	165 (78.57%)	0.848
Fully vaccinated/Booster doses (2/3 doses)	11 (1.42%)	2 (0.76%)	9 (1.77%)	1.000
Unknown	46 (8.55%)	10 (5.70%)	36 (10.02%)	0.768
Clinical laboratory biomarkers, median (IQR)/mean ( ± SD)				
Ct-ORF1ab	20.22 (17.8–23.63)	20.11 (18.61–23.94)	20.23 (17.79–23.69)	0.594
Ct-N	20.65 (18.3–24.15)	20.65 (18.75–24.63)	20.65 (18.21–24.01)	0.511
WBC, 10^9^/L	5.41 (4.18–7.08)	7.94 (5.42–11.39)	5.13 (4.01–6.66)	<0.001
NEUT, 10^9^/L	3.63 (2.44–5.68)	6.23 (3.80–8.97)	3.25 (2.27–4.59)	<0.001
NEUT, %	68.22 ± 14.51	78.02 ± 13.02	65.74 ± 18.83	<0.001
LYM, 10^9^/L	1.05 (0.73–1.45)	1.05 (0.60–1.39)	1.05 (0.75–1.46)	0.319
LYM, %	19.65 (11.8–30.28)	11.50 (8.15–19.80)	21.60 (13.90–31.65)	<0.001
Hemoglobin, g/L	117.43 ± 21.40	112.42 ± 22.91	118.70 ± 20.86	0.056
D-dimer, mg/L	1.05 (0.63–2.04)	1.78 (1.07–4.21)	0.90 (0.56–1.79)	<0.001
Interleukin-6, pg/mL	44.60 (25.90–149.18)	69.30 (27.94–164.05)	43.65 (24.70–137.33)	0.113
Procalcitonin, ng/mL	0.075 (0.023–0.229)	0.241 (0.097–0.947)	0.06 (0.02–0.13)	<0.001
CRP, mg/L	21.45 (7.11–63.99)	72.03 (12.97–146.63)	16.42 (5.55–50.24)	<0.001
SAA, mg/L	74.81 (23.88–305.76)	288.22 (49.87–320.00)	58.40 (19.99–219.29)	<0.001
Myoglobin, ng/L	96.24 (66.70–229.48)	161.80 (82.99–567.65)	92.24 (63.77–166.70)	<0.001
CK-MB, ng/L	3.01 (1.83–4.86)	4.27 (2.34–7.43)	2.77 (1.77–4.22)	<0.001
NT-proBNP, pg/mL	1,022.00 (413.58–2,504.00)	2,054.00 (890.40–5,621.00)	892.40 (353.60–2,134.00)	<0.001
TP, g/L	59.49 ± 5.95	59.77 ± 5.88	58.39 ± 6.20	0.145
AST, U/L	16.42 (10.79–24.37)	16.83 (10.66–24.63)	16.14 (10.78–24.48)	0.775
ALT, U/L	27.81 (21.20–40.29)	33.66 (21.53–49.65)	26.62 (21.20–37.65)	0.047
eGFR	80.00 (47.50–107.00)	61.00 (34.00–97.00)	83.00 (55.00–107.50)	0.012
BUN, mmol/L	8.12 (5.80–11.98)	10.64 (7.32–16.13)	7.64 (5.59–10.76)	<0.001
sCr, μmol/L	75.20 (55.80–106.45)	87.80 (61.30–161.20)	72.65 (55.63–100.80)	0.041
Therapy				
Antivirus(plaxlovid)	238 (90.49%)	52 (98.11%)	186 (88.57%)	0.035
Heparin	213 (80.99%)	48 (90.57%)	165 (78.57%)	0.051
Glucocorticoids	67 (25.48%)	39 (73.58%)	28 (13.33%)	<0.001
Transnasal high flow oxygen therapy	19 (7.22%)	12 (22.64%)	7 (3.33%)	<0.001
Non-invasive ventilation	48 (18.25%)	35 (66.04%)	13 (6.19%)	<0.001
Invasive mechanical ventilation	20 (7.60%)	19 (35.85%)	1 (0.48%)	<0.001

Values were presented as mean ± SD for continuous variables with a normal distribution, or median (IQR) for continuous variables without a normal distribution.

Ct-ORF1ab, Cycle Threshold-ORF1ab gene; Ct-N, Cycle Threshold-N gene; WBC, white blood cell; NEUT, neutrophil; LYM, lymphocytes; CRP, C-reactive protein; SAA, serum amyloid A; CK-MB, creatinine kinase-myocardial band; NT-proBNP, N-terminal pro-B-type natriuretic peptide; TP, total protein; LDH, lactate dehydrogenase; AST, aspartate aminotransferase; ALT, alanine aminotransferase; eGFR, glomerular filtration rate (estimated); BUN, blood urea nitrogen; sCr, serum creatinine.

The cutoffs for laboratory examinations: WBC: 3.5–9.5 × 10^9^/L; Neut: 1.8–6.3 × 10^9^/L; Neut%: 40%–75%; LYM: 1.1–3.2 × 10^9^/L; LYM%: 20%–50%; hemoglobin: 120–150 g/L; D-Dimer ≤ 0.5 mg/L; Interleukin-6 ≤ 6.6 pg/mL; Procalcitonin < 0.5 ng/mL; CRP: 0–6 mg/L; SAA: 0–10 mg/L; Myoglobin: male: 26.56–72.48 ng/L, female: 24.24–57.57 ng/L; CK-MB: male: ≤4.88 ng/L, female: ≤3.63 ng/L; NT-proBNP < 900 pg/mL; Tp: 65–85 g/L; Potassium: 3.5–5.3 mmol/L; Sodium: 137–147 mmol/L; Chlorate: 99–110 mmol/L; LDH: 120–250 U/L; ALT: male:9–50 U/L, female: 7–40 U/L; AST: male: 15–40 U/L, female: 13–35 U/L; eGFR: 90–120; BUN: male: 3.6–9.5 mmol/L, female: 3.1–8.8 mmol/L; sCr: male: 57–111 μmol/L, female: 41–81 μmol/L.

### Predictor screening and construction of the prognosis model

We collected 24 independent variables, including age, gender, and all laboratory biomarkers in **Table 1**. We applied a 7:3 nonrepetitive random sampling approach to the original dataset, partitioning it into the training and validation sets. To assess the validity of this division and detect any potential biases in data distribution, we performed a series of comparisons between the training and validation cohorts (**Supplementary Table S1**). None of the comparisons yielded *p*-values <0.05, indicating no statistically significant differences between the two cohorts.

**Figure 2:**
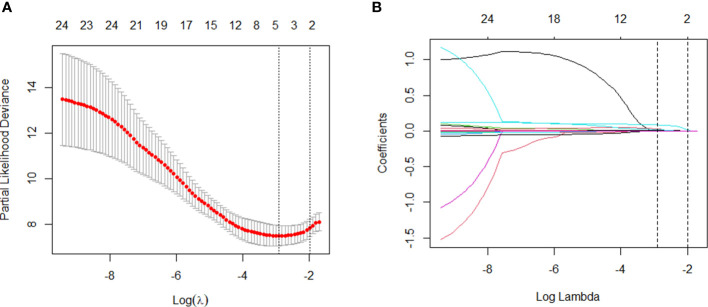
Variable selection: LASSO multiple logistic regression model. **(A)** After verifying the best parameter (λ) in the LASSO model, we draw a partial likelihood deviation (binomial deviation) curve and pair number (λ), and draw a vertical dashed line based on 1 SE. **(B)** By deriving the best λ, four variables with nonzero coefficients were selected.

**Figure 3:**
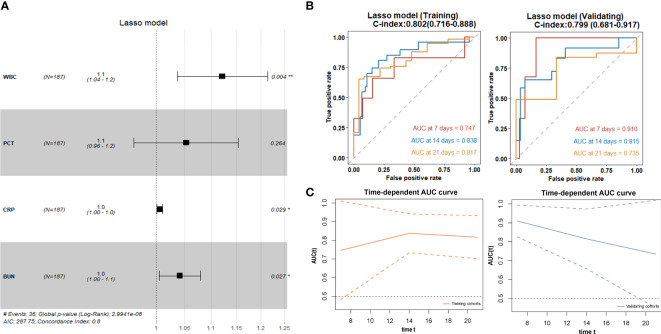
LASSO regression for selection of variables and discrimination performance. **(A)** The forest plot displaying Cox regression results of the LASSO model as HR values with 95% CI and *p*-values. **(B)** ROC curves of the LASSO model with AUROC and C-indexes with 95% CI in training and validating cohorts. Red: the 7-day ROC curve; blue: the 14-day ROC curve, yellow: the 21-day ROC curve. **(C)** Time-depentdent AUC curves of the LASSO model in training and validating cohorts.

We used these 24 independent variables for LASSO regression analysis. In the context of LASSO regression (**Figures 2A, B**), we identified WBC, PCT, CRP, and BUN as the variables with nonzero coefficients when the partial-likelihood deviance reached its minimum. We then included these variables in the Cox regression model, which we called the LASSO model (**Figure 3A**).

### Model validation

**Figure 4:**
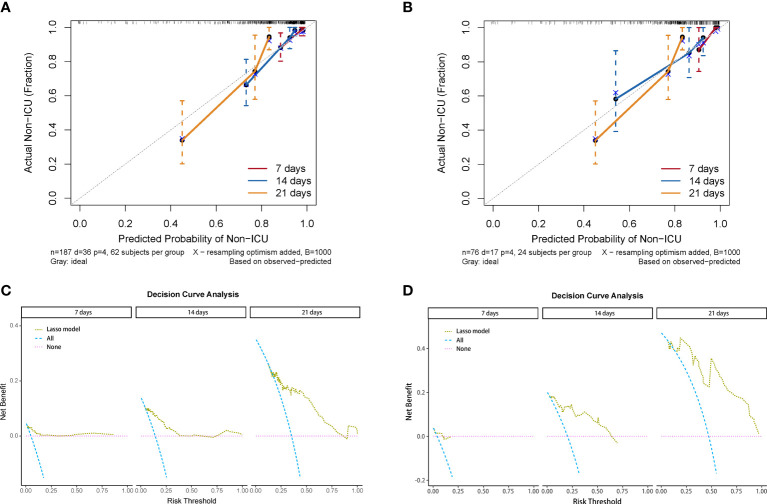
Calibration curve and decision curve analysis of the LASSO model. **(A)** Calibration plots of 7, 14, and 21 days displaying the relationship between predicted NIA probabilities and actual NIA proportions in training cohorts. **(B)** Calibration plots of 7, 14, and 21 days in validating cohorts. **(C)** Decision curves of the LASSO model showing the net benefit under different threshold probabilities in 7, 14, and 21 days in training cohorts. Yellow: LASSO model; blue: all patients receiving treatment; pink: no patient receiving treatment. **(D)** Decision curves of the LASSO model showing the net benefit under different threshold probabilities in 7, 14, and 21 days in validating cohorts.

We used the four variables selected by LASSO regression to build the final Cox regression model (**Figure 3A**). Concurrently, the performance of the LASSO model was also evaluated. We used the ROC curve to assess the model’s accuracy in predicting the risk of COVID-19 disease progression. The LASSO model showed good performance, with C-indexes of 0.802 and 0.799 in the training and validation cohorts, respectively. The AUROC values at 7, 14, and 21 days were also above 0.7 (**Figures 3B, C**), indicating its reliable discriminatory ability. The time-dependent AUC values were consistently above 0.7 as well, confirming the model’s excellent discrimination (**Figure 3C**). The LASSO model had the optimal predictive value on day 16. The calibration plot showed a close agreement between the predicted no ICU admission (NIA) probabilities and the observed NIA proportions, verifying the model’s accuracy in both the training and validation sets (**Figures 4A, B**). We presented the DCA for the LASSO model in **Figures 4C, D**. Over time, from 7 days to 14 days and then to 21 days, the threshold probability ranges with positive net benefit increased. In the 21-day DCA, the ranges in the training and validation cohorts were 0.2 to 0.8 and nearly 0.2 to 1, respectively. If the patient’s threshold probability is within these ranges, using the LASSO model to predict ICU admission provides more benefit than either the treat-all-patients scheme or the treat-none scheme. Since this model was built based on imputed data, we validated the model with the data before imputation (**Supplementary Figure S1**). The ROC curve and calibration plot showed that the model still had good discrimination and calibration.

### Model specification

**Figure 5:**
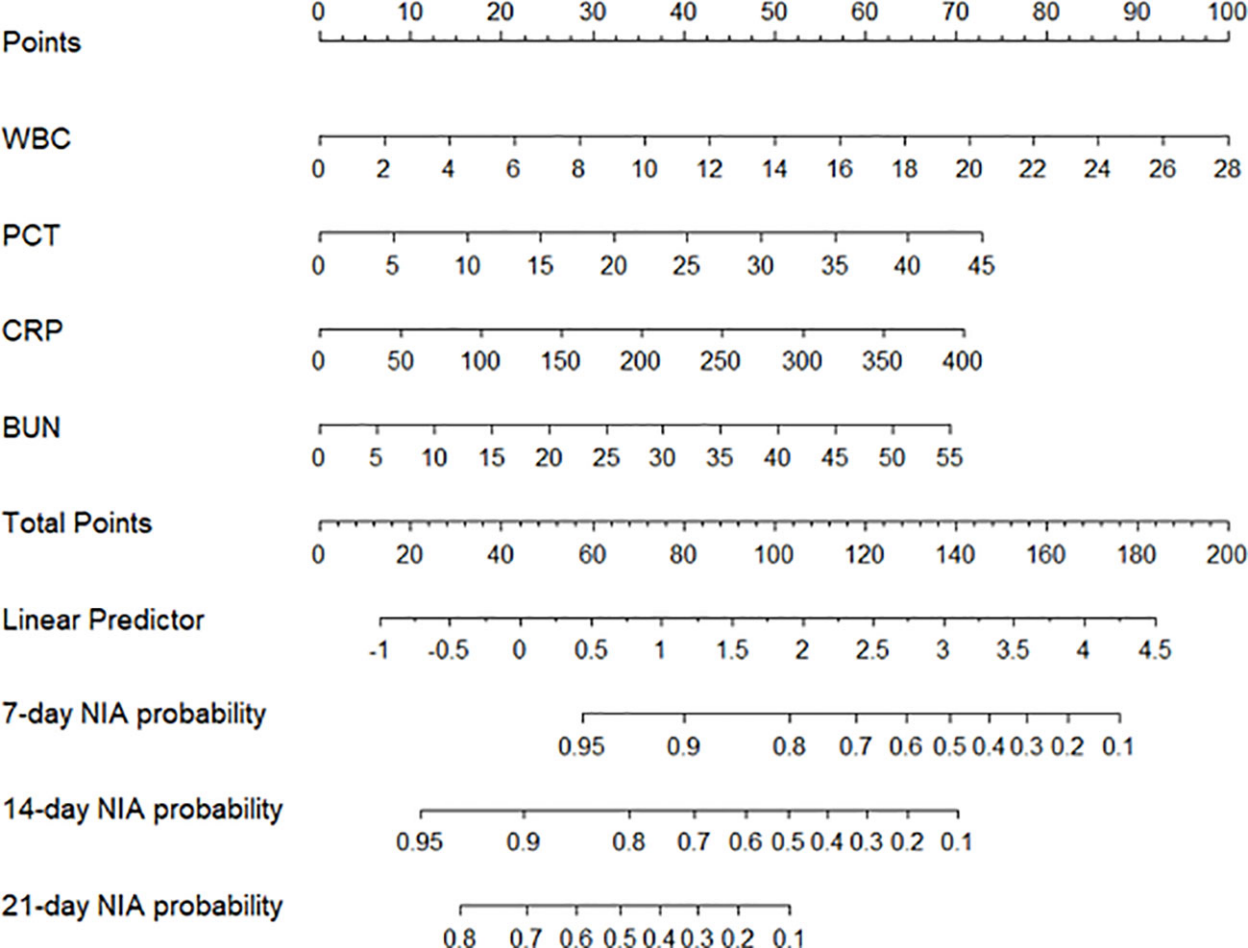
The nomogram of the LASSO model. Values in the scale ruler of each variable corresponded to their points in the first line. A summary of these points was displayed as the total points, and the total points corresponded to a patient’s NIA probability in 7, 14, and 21 days.

**Figure 6:**
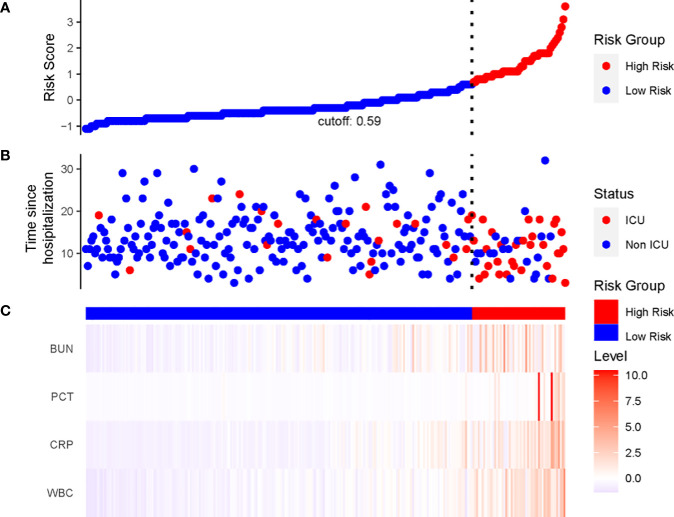
The linkage diagram of risk factors of the LASSO model. **(A)** The *Y* axis displayed the detailed risk score of each patient. **(B)** The Y axis displayed the follow-up time since admission along with patients’ outcomes. **(C)** Heatmap showing the standardized level of each variable in all patients.

We constructed a nomogram based on the Cox regression analysis of the LASSO model. This nomogram allows the accurate calculation of the NIA probability at 7, 14, and 21 days after hospitalization (**Figure 5**). The figure shows the non-ICU patients as blue dots and the ICU patients as red dots. The risk score has a dotted line that indicates the cutoff value. Patients to the left of the dotted line are classified as the low-risk score group, while those to the right are classified as the high-risk score group (**Figures 6A, B**). Moreover, the high-risk group had higher values of WBC, CRP, PCT, and BUN, indicating a positive correlation between risk scores and the probability of ICU admission (**Figure 6C**).

## Discussion

Despite the reports that most of the Omicron-infected patients were asymptomatic or mild cases during the Omicron variant pandemic, the elderly population had significantly higher rates of severe/critical infection and mortality than the rest of the population (26). Geriatric patients with multiple comorbidities were more prone to have serious outcomes. Cardiovascular injury was a major risk factor for developing severe infection (27, 28). Throughout the COVID-19 pandemic, many studies have shown that patients with concurrent MI were more prone to requiring ICU care than those without MI (29). As the Omicron variant spreads rapidly and many countries are relaxing their strict COVID-19 management measures, Omicron variant infections are becoming more prevalent, resulting in a spike of cases. Therefore, it is essential to identify and prevent potential severe cases, especially for the high-risk groups. In this study, we analyzed the clinical features and developed a prognostic model based on laboratory biomarkers to early predict the probability of patients with Omicron variant infection and MI progressing to a severe condition.

During the outbreak of wild-type SARS-CoV-2, biomarkers that have been clinically validated or newly discovered have the potential to predict the severity and prognosis of COVID-19 patients. Pier Paolo Sainaghi and colleagues pointed out that among several cytokines and chemokines analyzed, IP-10 was positively associated with increased disease severity and poor prognosis, but negatively associated with faster recovery. Baseline serum levels of IP-10 and CRP after 7 days in the hospital independently predicted disease progression (30). Novel bioanalytes, such as Growth Arrest-Specific 6 (Gas6), a secreted glycoprotein that played a key role in regulating immune homeostasis, fibrosis, and thrombosis, were also found to have prognostic value. Plasma levels above 58.0 ng/mL indicated a higher risk of severe disease progression (31). Moreover, a prospective observational study showed that reduced levels of Gas6 and its soluble receptors, especially sAxl, were related to a history of post-COVID-19 hair loss (32). In summary, these findings highlight the potential of both established and novel biomarkers in improving our knowledge and management of COVID-19 outcomes.

In our prognostic model, we identified higher levels of WBC, PCT, CRP, and BUN as significant factors that increased the probability of ICU admission. WBCs are the main immune cells in the body, involved in combating pathogen invasion and facilitating immune reactions. In Omicron infection, changes in WBC count may be related to the severity and prognosis of the disease. There is also an association between increased WBC and MI. This may be due to the inflammatory response induced by MI, which activates the immune system. PCT has a role in MI and the prognosis of COVID-19. PCT is a prohormone produced by the thyroid C cells, and it is normally present in very low levels in the blood. However, during MI, especially when associated with infection, inflammation, or trauma, PCT levels may rise. Higher levels of PCT may indicate the severity of myocardial damage and the patient’s condition. PCT has been widely studied in the prognosis of COVID-19. Some studies have found that elevated PCT levels are associated with the severity of infection, inflammatory response, and tissue damage in COVID-19 (33–35). Higher PCT levels may also indicate worsening infection and poor prognosis (36, 37). In patients with COVID-19 infection or MI, the levels of CRP often increase (38). This may be because MI triggers an inflammatory response, activating the immune system and leading to more CRP production. By measuring the levels of CRP in the blood, the degree of inflammation and prognosis in patients with COVID-19 infection and MI can be assessed (35, 38). In the study of 182 COVID-19 patients by Li et al., WBC, hs-C-reactive protein, and PCT were independently associated with MI in a multivariable adjusted analysis (39). Furthermore, elevated BUN levels were observed, suggesting potential renal function impairment or dehydration, which can be associated with a poorer prognosis (40–42). The pathogenic mechanisms leading to MI, such as reduced cardiac output and subsequent renal hypoperfusion, could elucidate the observed increase in BUN levels (43, 44). Consequently, monitoring BUN levels could provide valuable information about renal health and the overall hemodynamic status of the patient.

The predictors used in this study were obtained from the patients’ admission data, enabling the early identification of potentially severe cases at the start of hospitalization.

This proactive approach enables the prompt initiation of suitable treatment interventions. Additionally, it facilitates the efficient allocation of medical resources, thus enhancing their utilization for optimal patient care. In our study, we focused on laboratory biomarkers as our model’s predictors, because laboratory biomarkers are objective and impartial, and in emergency situations such as when patients are unconscious or unable to communicate their medical condition clearly, laboratory biomarkers can quickly, easily, and accurately indicate the patient’s status. Furthermore, the evaluation of these four predictors is cost-effective and feasible.

### Limitations

Our study has several limitations that should be acknowledged. Firstly, this is a single-center study that only included elderly patients from a designated hospital in Shanghai, which may affect the generalizability of our findings to other settings and populations. The incomplete characterization of the population, such as the details of pre-hospitalization treatment, prior infection status, and other factors, may also limit the validity of the findings. To obtain more robust and comprehensive scientific analyses, it would be desirable to include multiple centers and participants with diverse and more complete population characteristics and health conditions. Secondly, the missing data in laboratory examinations and viral shedding time may introduce potential biases in data analysis and interpretation. Thirdly, the model of this study may not be fully applicable to all patients infected with the Omicron variant and complicated with MI, such as outpatients in isolation sites or communities, because all participants involved in this study were hospitalized patients.

## Conclusion

We developed and validated a prognostic model based on four laboratory biomarkers—WBC, CRP, PCT, and BUN—to predict the severity of Omicron variant infection complicated with MI. This prognostic model demonstrated superior discriminatory ability, calibration, and net benefit, indicating its high potential for clinical application. This study contributed to further refine and identify the “high-risk population” among elderly individuals with infection and associated complications, who are prone to severe disease progression. This model can alert healthcare professionals to provide timely and appropriate care and treatment to these individuals.

## Data availability statement

The raw data supporting the conclusions of this article will be made available by the authors, without undue reservation.

## Ethics statement

The studies involving humans were approved by the Ethics Committee of the Fourth People’s Hospital of Tongji University. The studies were conducted in accordance with the local legislation and institutional requirements. The human samples used in this study were acquired from a by-product of routine care or industry. Written informed consent for participation was not required from the participants or the participants’ legal guardians/next of kin in accordance with the national legislation and institutional requirements.

## Author contributions

XY: Data curation, Formal analysis, Writing – original draft. XL: Data curation, Formal analysis, Writing – original draft. SX: Formal analysis, Methodology, Writing – original draft. TL: Formal analysis, Methodology, Visualization, Writing – original draft. MZ: Data curation, Investigation, Writing – original draft. CS: Conceptualization, Methodology, Supervision, Writing – review & editing. QM: Funding acquisition, Project administration, Resources, Supervision, Writing – review & editing. LX: Funding acquisition, Project administration, Resources, Supervision, Writing – review & editing.
