# Supplementary material for: Development and validation of a prognostic model based on clinical laboratory biomarkers to predict admission to ICU in Omicron variant-infected hospitalized patients complicated with myocardial injury
Source: Front Immunol. 2024 Feb 1;15:1268213. doi: 10.3389/fimmu.2024.1268213 (PMC10868580; doi:10.3389/fimmu.2024.1268213)
Supplement: Supplementary file 1 [file DataSheet_1.docx]

| **Variables** | **Total (n = 263)** | **The Training** **cohort (n = 187)** | **The Validating cohort (n = 76)** | ***P-*value^a^** |
| --- | --- | --- | --- | --- |
| ICU (n) |  |  |  | 0.568 |
| Non-ICU group | 210 | 151 | 59 |  |
| ICU group | 53 | 36 | 17 |  |
| Age (year) | 87 (61-104) | 87 (61-104) | 88 (62-100) | 0.408 |
| Gender (n) |  |  |  | 0.827 |
| Male | 108 | 76 | 32 |  |
| Female | 155 | 111 | 44 |  |
| Hypertension | 184 | 132 | 52 | 0.728 |
| Diabetes | 57 | 42 | 15 | 0.627 |
| Coronary artery disease | 117 | 80 | 37 | 0.383 |
| Ct-ORF1ab | 20.21 (17.80-23.55) | 20.37 (17.70-23.50) | 20.10 (18.21-24.18) | 0.969 |
| Ct-N | 20.65 (18.30-24.10) | 21.01 (18.30-24.29) | 20.41 (18.36-23.87) | 0.958 |
| WBC, 10^9/L | 5.39 (4.16-7.08) | 5.31 (4.16-7.00) | 5.72 (4.11-7.32) | 0.468 |
| NEUT, 10^9/L | 3.62 (2.42-5.68) | 3.52 (2.59-5.24) | 3.84 (2.29-6.02) | 0.582 |
| NEUT, % | 69.40 (58.30-78.60) | 68.80 (58.40-77.50) | 69.90 (57.40-81.70) | 0.422 |
| LYM, 10^9/L | 1.05 (0.73-1.45) | 1.03 (0.73-1.43) | 1.08 (0.71-1.48) | 0.832 |
| LYM, % | 19.60 (11.50-30.20) | 19.20 (12.20-30.50) | 20.40 (10.35-30.15) | 0.855 |
| Hemoglobin, g/L |  | 118.70 ± 21.04 | 114.08 ± 22.00 | 0.227 |
| D-dimer, mg/L | 1.06 (0.64-2.07) | 1.02 (0.61-1.98) | 1.19 (0.72-2.24) | 0.213 |
| Interleukin-6, pg/ml | 44.54 (25.62-148.50) | 43.37 (24.48-120.00) | 47.85 (26.86-191.95) | 0.091 |
| Procalcitonin, ng/mL | 0.079 (0.022-0.236) | 0.074 (0.022-0.229) | 0.095 (0.022-0.263) | 0.707 |
| CRP, mg/L | 21.54 (7.39-64.44) | 20.76 (6.74-62.11) | 26.39 (9.30-78.5) | 0.353 |
| SAA, mg/L | 74.36(23.67-299.05) | 72.30 (21.95-291.41) | 79.64 (28.04-317.28) | 0.586 |
| Myoglobin, ng/L | 96.23(66.37-227.10) | 98.20 (65.74-242.30) | 92.91 (67.55-175.45) | 0.607 |
| CK-MB, ng/L | 3.00 (1.83-4.85) | 3.15 (1.90-5.24) | 2.44 (1.76-4.41) | 0.159 |
| NT-proBNP, pg/mL | 1038.00 (409.80-2851.00) | 1014.00 (397.40-2815.00) | 1419.00 (484.60-2919.50) | 0.442 |
| TP, g/L | 59.39 (55.75-63.34) | 59.67 (55.96-63.69) | 58.98 (55.71-62.18) | 0.354 |
| AST, U/L | 16.58 (10.79-24.54) | 16.31 (11.34-24.26) | 16.83 (10.08-27.74) | 0.936 |
| ALT, U/L | 27.78 (21.47-40.27) | 28.08 (21.93-41.77) | 27.26 (19.61-36.77) | 0.294 |
| eGFR | 80.00(47.00-105.00) | 78.00 (47.00-104.00) | 82.00 (46.25-112.75) | 0.632 |
| BUN, mmol/L | 8.17 (5.86-12.11) | 8.15 (5.86-12.02) | 8.56 (5.74-12.61) | 0.805 |
| sCr, umol/L | 75.20 (55.80-108.70) | 76.00 (55.80-109.50) | 73.60 (55.88-101.45) | 0.825 |

**Table S1. Comparisons between training and validating cohorts**

For continuous variables that followed a normal distribution, an unpaired t-test (two-tailed) was employed. For continuous variables that did not exhibit a normal distribution, the Wilcoxon signed-rank test (two-tailed) was used. Pearson's chi-squared test (two-tailed) was utilized for categorical variables.

**
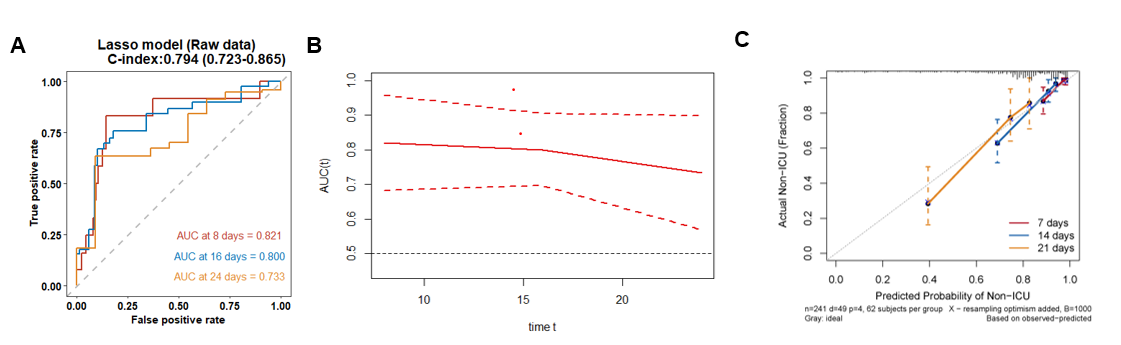
Figure S1. The discrimination and calibration performance of final model in raw data.**

(A) ROC curves of the Lasso model with AUROC and C-indexes with 95% CI in raw data. Red: the 7-day ROC curve; blue: the 14-day ROC curve, yellow: the 21-day ROC curve. (B) Time-depentdent AUC curves of the final model in raw data. (C) Calibration plots of 7, 14, and 21 days displaying the relationship between predicted NIA probabilities and actual NIA proportions in raw data. Raw data: the data set that combine the training and the validation data set.
